# Does autoimmune disease impair the survival of hepatocellular carcinoma patients undergoing liver resection? A multi-institutional observational study

**DOI:** 10.1007/s00432-024-05885-1

**Published:** 2024-07-20

**Authors:** Chao-Wei Lee, Hsing-Yu Chen, Ping-Han Tsai, Wei-Chen Lee, Chih-Chi Wang, Ming-Chin Yu, Chun-Wei Chen, Po-Ting Lin, Bo-Huan Chen, Sheng-Fu Wang, Pei-Mei Chai, Hsin-I. Tsai

**Affiliations:** 1 Division of General Surgery, Department of Surgery, Linkou Chang Gung Memorial Hospital, Guishan, Taoyuan, Taiwan; 2 College of Medicine, Chang Gung University, Guishan, Taoyuan, Taiwan; 3 Graduate Institute of Clinical Medical Sciences, Chang Gung University, Guishan, Taoyuan, Taiwan; 4 Division of Chinese Internal Medicine, Center for Traditional Chinese Medicine, Taoyuan Chang Gung Memorial Hospital, Guishan, Taoyuan, Taiwan; 5 School of Traditional Chinese Medicine, College of Medicine, Chang Gung University, Guishan, Taoyuan, Taiwan; 6 Division of Rheumatology, Allergy and Immunology, Department of Internal Medicine, New Taipei Municipal Tucheng Hospital, Tu-Cheng, New Taipei City, Taiwan; 7 Division of General Surgery, Department of Surgery, Kaohsiung Chang Gung Memorial Hospital, Kaohsiung, Taiwan; 8 Department of Surgery, New Taipei Municipal Tu-Cheng Hospital (Built and Operated by Chang Gung Medical Foundation), Tu-Cheng, New Taipei City, Taiwan; 9 Department of Gastroenterology and Hepatology, Linkou Chang Gung Memorial Hospital, Guishan, Taoyuan, Taiwan; 10 Department of Nursing, Linkou Chang Gung Memorial Hospital, Guishan, Taoyuan, Taiwan; 11 Department of Anesthesiology, Linkou Chang Gung Memorial Hospital, No. 5, Fuxing St., Guishan Dist., 33305 Taoyuan, Taiwan

**Keywords:** Autoimmune disease, Chang Gung Research Database, Hepatocellular carcinoma, Liver resection

## Abstract

**Background:**

Patients with autoimmune diseases (AD) generally carry an increased risk of developing cancer. However, the effect of AD in hepatocellular carcinoma (HCC) patients receiving surgical treatment is uncertain. The present study aimed to investigate the potential influence of AD on the survival of HCC patients undergoing hepatectomies.

**Methods:**

Operated HCC patients were identified from the Chang Gung Research Database, and the survival outcomes of HCC patients with or without AD were analyzed ad compared. Cox regression model was performed to identify significant risk factors associated with disease recurrence and mortality.

**Results:**

From 2002 to 2018, a total of 5532 patients underwent hepatectomy for their HCC. Among them, 229 patients were identified to have AD and 5303 were not. After excluding cases who died within 30 days of surgery, the estimated median overall survival (OS) was 43.8 months in the AD (+) group and 47.4 months in the AD (–) group (*P* = 0.367). The median liver-specific survival and disease-free survival (DFS) were also comparable between the two groups. After Cox regression multivariate analysis, the presence of AD did not lead to a higher risk of all-cause mortality, liver-specific mortality, or disease recurrence.

**Conclusion:**

Our study demonstrated that autoimmune disease does not impair the OS and DFS of HCC patients undergoing liver resections. AD itself is not a risk factor for tumor recurrence after surgery. Patients eligible for liver resections, as a result, should be considered for surgery irrespective of the presence of AD. Further studies are mandatory to validate our findings.

## Introduction

Hepatocellular carcinoma (HCC) is the most common primary malignancy of the liver and ranked the 6th most common cancer worldwide in 2020 (Sung et al. 2021). It is a primary tumor of the liver that usually develops from chronic liver disease, particularly in patients with cirrhosis secondary to alcohol abuse and chronic hepatitis B or C virus infections. In addition to well-known etiological factors, recent studies have demonstrated that dietary as well as genetic factors may also predispose to the development and progression of various cancers, including HCC (Huang et al. 2024; Huang et al. 2023). The comprehension of causal and mechanistic relationships has led to the development and evolution of novel targeted therapies against these lethal malignancies (Huang et al. 2024; Huang et al. 2023a; Huang et al. 2023b). In the past few decades, moreover, various observational studies have also investigated the relationship between autoimmune liver disease and HCC. Literature has demonstrated that patients with autoimmune liver diseases, including autoimmune hepatitis (AIH) (Tansel et al. 2017; Valean et al. 2019), primary biliary cholangitis (PBC) (Giannini et al. 2022; Sy et al. 2022), and primary sclerosing cholangitis (PSC) (Bosch et al. 2021) are at increased risk to develop hepatobiliary malignancies (Rigopoulou et al. 2021). However, the relationship between HCC and other autoimmune diseases is still controversial.

Patients with autoimmune diseases (AD) generally carry an increased risk of developing cancer. Recent epidemiologic evidence has suggested that systematic lupus erythematosus (SLE) was a risk factor for digestive cancers, hematologic cancers, urological cancers in addition to cancer in lung, larynx, cervical, vagina/vulva, renal, bladder, skin and thyroid (Zhang et al. 2022; Bae et al. 2019). Numerous studies also have found that patients with polymyositis (PM) and dermatomyositis (DM) are also at an increased risk for developing cancers and are associated with high mortality (Wakata et al. 2002; DeWane et al. 2020). Some other studies have shown an increased risk of hepatitis B virus (HBV) or hepatitis C virus (HCV) infections in patients with rheumatoid arthritis (RA) (Kojima et al. 2002; Hsu et al. 2018), which in turn may increase the risks of developing HCC for RA patients. On the contrary, a few studies demonstrated that RA was associated with a declined possibility of HCC for East Asians (Zhang et al. 2023; Hsu 2018). The relationship between AD and HCC is complex and the management of AD in patients with cancer becomes challenging. Considering the complexity of treatment to HCC and AD, it is crucial to understand the effect of AD in HCC patients receiving surgical treatment. As the evidence in patients with AD and their prognosis after liver resections for HCC is still lacking, we aim to investigate the potential influence of AD on the survival of HCC patients undergoing hepatectomies.

## Materials and methods

### Data source

The Chang Gung Research Database (CGRD), which collected the clinical data from eight Chang Gung memorial hospitals (CGMH) in Taiwan since year 2000, was the primary data source of the current study. With more than 10,070 beds and 500,000 emergency visits each year, the CGRD has accounted for 12.4% of inpatients and 21.2% of outpatients in Taiwan and become an excellent database for clinical studies (Liu et al. 2020; Lee et al. 2021). For cancer patients, it contains comprehensive cancer registry maintained in a prospective manner. The information is manually validated with a high completeness rate (Chiang et al. 2015; Chiang 2019). Both the International Classification of Diseases, 9th and 10th revision, Clinical Modification (ICD-9-CM and ICD-10-CM) codes and the International Classification of Diseases for Oncology, 3rd edition (ICD-O-3) are used in the CGRD. For individual privacy, the patient identity is protected by encryption. The medical information is prospectively digitalized and stored in the CGRD and is amenable for investigators to perform large-scale clinical analysis (Lee et al. 2021; Lee 2022).

### Study design and population

**Fig. 1:**
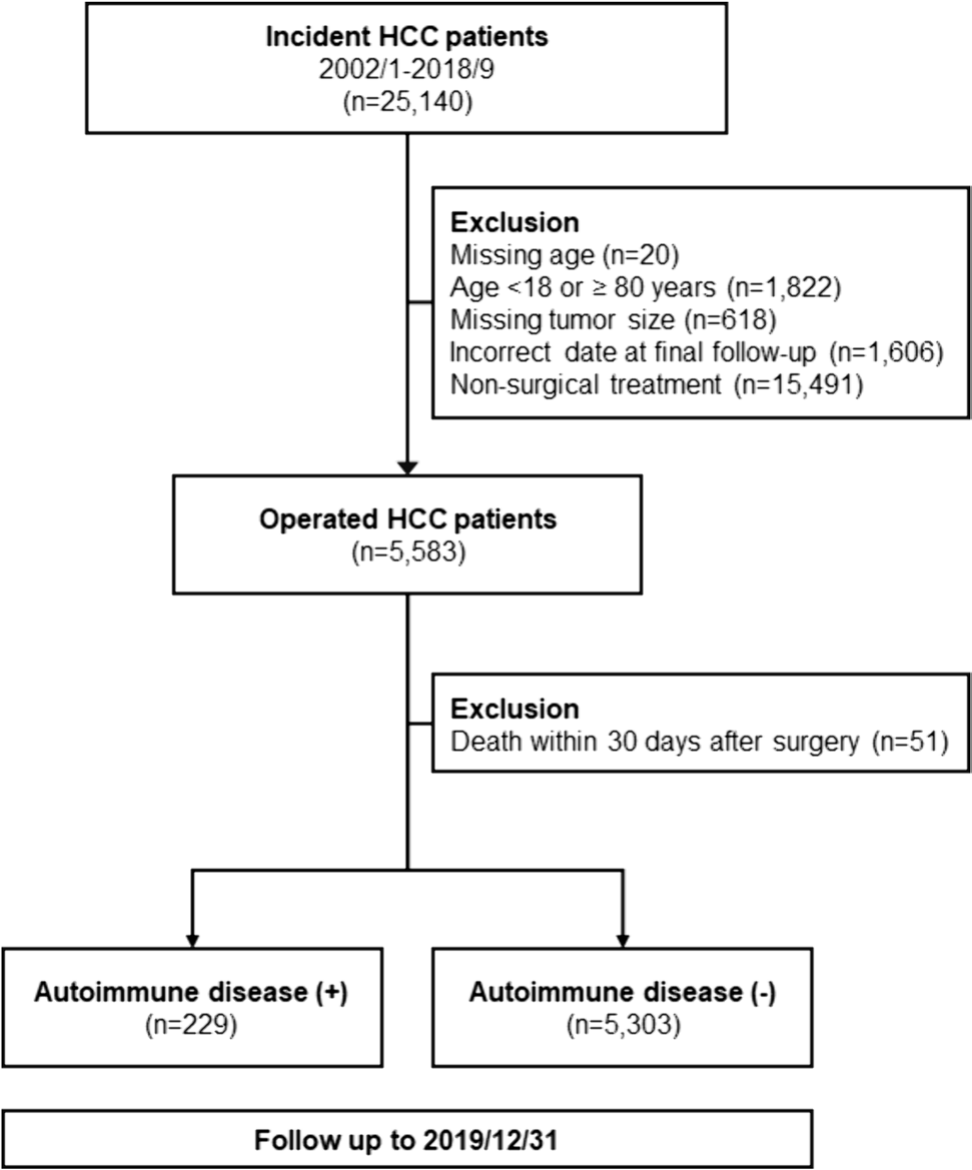
Flow diagram of the current study. HCC patients diagnosed from 2002 to 2018 were retrieved from the CGRD database (*n* = 25,140). Those who received non-surgical treatment, who had missing critical data, or who died within 30 days of surgery were excluded from further analysis. The survival outcomes of HCC patients with or without autoimmune diseases (AD (+) and (AD (–), respectively) were then analyzed ad compared

Figure 1 is the flowchart of the current study. The ICD-9-CM code 1550 and ICD-10-CM code C220 were adopted to identify HCC patients from the CGRD. Patients who received curative liver resection from January 2002 to September 2018 were enrolled as the study population. Those who received non-surgical treatment, who had missing data, who aged younger than 18 or older than 80 years, or who died within 30 days of surgery were excluded from further analysis. Tumors were staged according to the 8th edition of AJCC TNM staging system for HCC in the current study (Edge et al. 2017; Amin et al. 2017). To investigate the influence of AD on the survival of HCC patients undergoing hepatectomies, HCC patients were further examined for the diagnosis of AD (Table 1). The survival outcomes of HCC patients with AD (AD (+)) or without AD (AD (–)) were then analyzed ad compared. This study was approved by the Institutional Review Boards of CGMH (IRB No.: 201900800B0).

**Table 1:** The types of autoimmune diseases

Types of autoimmune diseases	Patient number (*n* (%))
Systemic lupus erythematosus	17 (7.4%)
Myositis^a^	158 (69.0%)
Sjogren syndrome	10 (4.4%)
Psoriasis	6 (2.6%)
Ankylosing spondylitis	13 (5.7%)
Vasculitis	18 (7.9%)
Pemphigus	1 (0.4%)

^a^ Including systemic myositis and dermatomyositis

### Outcome assessment and statistical analysis

Overall survival (OS) and liver-specific survival were the primary study outcomes while disease-free survival (DFS) was the secondary outcome of the current study. The first date of definite diagnosis for HCC was set as the index date. DFS defined the period between the index date and the date of the first documented clinical recurrence or the end of year 2019. Liver-specific survival spanned the period between the index date and the date of liver-cause mortality or the end of year 2019. The liver-causes included tumor recurrence, metastasis, and complications of decompensated liver cirrhosis. OS defined the period between the index date and the date of all-cause mortality or the end of year 2019.

The continuous variables were analyzed by Mann-Whitney U test, and chi-square statistics was employed to analyze categorical variables. Kaplan-Meier survival estimation with log-rank test was used to assess the OS, liver-specific survival, and DFS. Cox regression model was performed to identify significant risk factors associated with disease recurrence and mortality. The freeware Konstanz information miner (KNIME) and the commercial statistic software STATA (Stata Statistical Software: release 17. College Station, TX: Stata Corp LLC) were adopted to process and analyze the data (Berthold et al. 2009). All statistics with *P* < 0.05 were regarded as statistically significant.

## Results

### Patient characteristics

As shown in Fig. 1, a total of 5532 patients underwent hepatectomy for HCC treatment. Among them, 229 patients were identified to have AD and 5303 were not. The types of AD included in the current study were summarized in Table 1. Of the 229 patients, 17 patients had Systemic lupus erythematosus (SLE) (7.4%), 158 had myositis (69%), 10 had Sjogren syndrome (4.4%), 6 had psoriasis (2.6%), 13 had ankylosing spondylitis (5.7%), 18 had vasculitis (7.9%) and 1 had pemphigus (0.4%). Shown in Table 2, HCC patients were male-predominant with a median age of 60 years. When compared with patients without AD, those with AD were older (*P* = 0.045) with a higher female tendency (28.4% vs. 21.7%, *P* = 0.018). There was also a higher incidence of diabetes mellitus (DM), hypertension, and chronic HCV infection in the AD (+) group (all *P* < 0.001). No statistical significance was observed in the lifestyle such as cigarette smoking, alcohol consumption, or betelnut chewing between the two groups. The average tumor size of the entire cohort was 35.0 millimeters. The AD (+) group had a slightly smaller tumor size and less stage I tumors than the AD (–) group. Consistent with a higher incidence of DM and hypertension, there were more patients in the AD (+) group having taken metformin and aspirin (all *P* < 0.001). As for their biochemical profiles, AD (+) group appeared to have significantly lower hemoglobin level, platelet count and international normalized ratio (INR) than AD (–) group, but no significance was observed in serum levels of aspartate transaminases (AST), alanine transaminases (ALT), total bilirubin, prognostic nutritional index (PNI), platelet to lymphocyte ratio (PLR) or neutrophil to lymphocyte ratio (NLR).

**Table 2:** Baseline features of operated hepatocellular carcinoma (HCC) patients with or without autoimmune diseases (AD)

	All subjects (*n* = 5532^a^)	With AD (*n* = 229)	Without AD (*n* = 5303)	*P*-value
**Demographics**				
*Gender*				
Female	1218 (22.0%)	65 (28.4%)	1153 (21.7%)	0.018
Male	4314 (78.0%)	164 (71.6%)	4150 (78.3%)	
*Age (years)*	60.0 (51.0–67.0)	62.0 (53.0–67.0)	60.0 (51.0–67.0)	0.084
*Age group*				
Below 40	439 (7.9%)	10 (4.4%)	429 (8.1%)	0.045
41–60	2469 (44.6%)	96 (41.9%)	2373 (44.7%)	
61 and above	2624 (47.4%)	123 (53.7%)	2501 (47.2%)	
*Co-morbidities*				
Diabetes	1358 (24.5%)	90 (39.3%)	1268 (23.9%)	< 0.001
Hypertension	1931 (34.9%)	126 (55.0%)	1805 (34.0%)	< 0.001
Viral infection				< 0.001
HBV	2691 (48.6%)	79 (34.5%)	2612 (49.3%)	
HCV	1209 (21.9%)	73 (31.9%)	1136 (21.4%)	
HBV + HCV	304 (5.5%)	14 (6.1%)	290 (5.5%)	
*Lifestyles*				
Cigarette smoking	651 (11.8%)	21 (9.2%)	630 (11.9%)	0.21
Alcohol consumption	556 (10.1%)	24 (10.5%)	532 (10.0%)	0.83
Betel nut chewing	170 (3.1%)	9 (3.9%)	161 (3.0%)	0.44
**Disease severity**				
*Child-Pugh Score classification*				
A	2973 (97.9%)	118 (96.7%)	2855 (97.9%)	0.36
B	64 (2.1%)	4 (3.3%)	60 (2.1%)	
*Cirrhosis*				
No	1619 (51.5%)	53 (43.4%)	1566 (51.8%)	0.070
Yes	1526 (48.5%)	69 (56.6%)	1457 (48.2%)	
*Tumor size (mm)*	35.0 (23.0–60.0)	32.0 (21.0–55.0)	35.0 (23.0–60.0)	0.049
*TNM staging (AJCC 8th version)*				
I	2919 (53.8%)	111 (49.6%)	2808 (54.0%)	0.006
II	1433 (26.4%)	70 (31.3%)	1363 (26.2%)	
III	998 (18.4%)	35 (15.6%)	963 (18.5%)	
IV	73 (1.3%)	8 (3.6%)	65 (1.3%)	
*Medications*				
Anti-HCV/HBV therapy	439 (7.9%)	18 (7.9%)	421 (7.9%)	0.97
Metformin	334 (6.0%)	38 (16.6%)	296 (5.6%)	< 0.001
Aspirin	221 (4.0%)	28 (12.2%)	193 (3.6%)	< 0.001
*Biochemical profiles*				
α-Fetoprotein	15.8 (4.9-212.7)	10.2 (4.4-153.1)	16.1 (5.0-218.7)	0.098
Albumin	4.1 (3.7–4.4)	4.1 (3.7–4.4)	4.1 (3.7–4.4)	0.33
Hemoglobin	13.8 (12.4–14.9)	13.4 (11.8–14.7)	13.8 (12.4–14.9)	0.010
Platelet	174.0 (134.0-220.0)	164.0 (124.0-210.0)	174.0 (135.0-220.0)	0.017
INR	1.1 (1.0-1.1)	1.0 (1.0-1.1)	1.1 (1.0-1.1)	0.004
AST	39.0 (28.0–65.0)	41.0 (28.0–64.0)	39.0 (28.0–65.0)	0.77
ALT	40.0 (26.0–68.0)	36.0 (25.0–67.0)	40.0 (26.0–68.0)	0.22
Total bilirubin	0.8 (0.6–1.1)	0.8 (0.6-1.0)	0.8 (0.6–1.1)	0.60
PNI group				
Normal	1839 (44.6%)	74 (42.8%)	1765 (44.7%)	0.90
Mild	1077 (26.1%)	48 (27.7%)	1029 (26.1%)	
Mod to severe	704 (17.1%)	28 (16.2%)	676 (17.1%)	
Serious	501 (12.2%)	23 (13.3%)	478 (12.1%)	
PLR	15.6 (12.2–19.6)	16.0 (11.6–20.0)	15.6 (12.2–19.6)	0.57
ALBI group				
Grade 1(lowest risk)	2998 (65.3%)	123 (64.1%)	2875 (65.4%)	0.92
Grade 2	1508 (32.8%)	65 (33.9%)	1443 (32.8%)	
Grade 3(highest risk)	85 (1.9%)	4 (2.1%)	81 (1.8%)	
NLR	2.4 (1.6–4.8)	2.3 (1.7–4.1)	2.4 (1.6–4.8)	0.51

*AD* autoimmune disease, *ALBI* albumin-bilirubin grade, *ALT* Alanine aminotransferase, *AJCC* American Joint Committee on Cancer, *AST* Aspartate aminotransferase, *HBV* Hepatitis B virus, *HCV* Hepatitis C virus, *INR* international normalized ratio, *NLR* neutrophil to lymphocyte ratio, *PLR* platelet to lymphocyte ratio, *PNI* prognostic nutritional index

^a^Number excluded surgical mortality (30-d mortality)

### Survival outcome

**Fig. 2:**
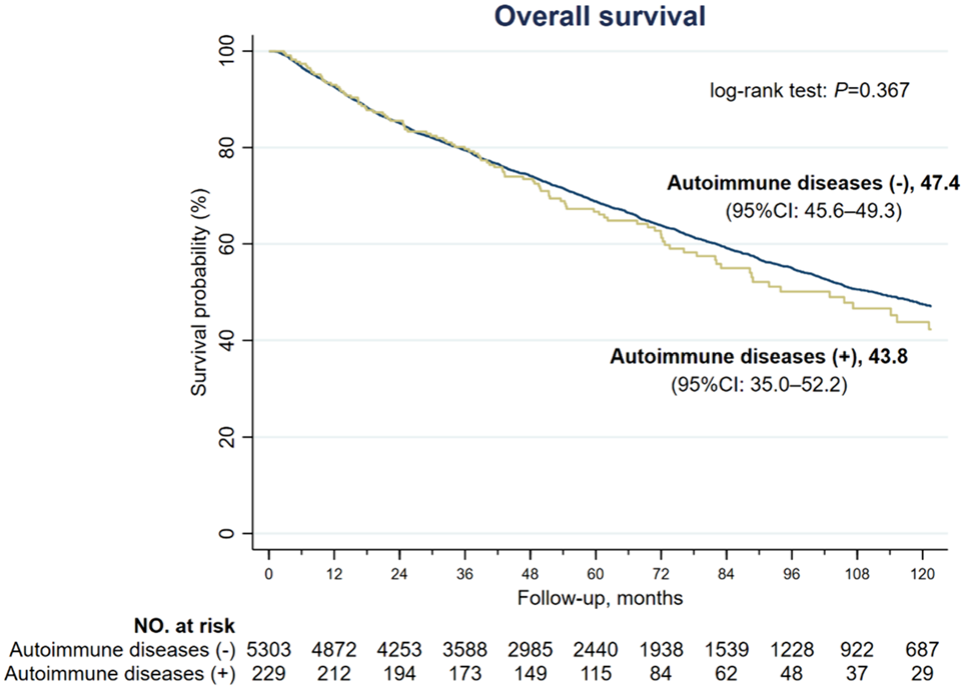
Kaplan-Meire overall survival (OS) curves of HCC patients without and without autoimmune diseases. The median OS was 43.8 months in the AD (+) group and 47.4 months in the AD (–) group (*P* = 0.367)

Among all 5532 patients, 51 patients had deceased within 30 days of surgery, among which, 49 in the AD (–) group and 2 in the AD (+) group, with no statistical difference. As shown in Fig. 2, the estimated median OS was 43.8 months (95% CI 35.0–52.2 months) in the AD (+) group and 47.4 months (95% CI 45.6–49.3 months) in the AD (–) group (*P* = 0.367). The 1-year, 3-year and 5-year overall survival rates were 92.7%, 79.4% and 68.7%, respectively, in the entire cohort and were comparable between AD (+) and AD (–) groups (Table 3). The 10-year survival rate was 47.3% in the entire cohort and was significantly lower in AD (+) than in AD (–) group (37.4% vs. 47.6%, *P* = 0.025).

**Table 3:** Survival outcome of operated hepatocellular carcinoma (HCC) patients with or without autoimmune diseases (AD)

	All subjects (*n* = 5532)	With AD (*n* = 229)	Without AD (*n* = 5303)	*P*-value
*Overall-survival (OS) rate (%)*				
1-year	92.7 (92.0–93.3)	89.0 (82.8–93.0)	92.8 (92.1–93.4)	0.090
3-year	79.4 (78.3–80.5)	74.9 (67.2–81.1)	79.6 (78.5–80.7)	0.163
5-year	68.7 (67.4–70.0)	64.4 (56.0–71.6)	68.9 (67.5–70.2)	0.169
10-year	47.3 (45.5–49.1)	37.4 (27.0–47.8)	47.6 (45.7–49.4)	0.025
*Liver-specific survival rate (%)*				
1-year	93.9 (93.2–94.6)	90.4 (84.1–94.3)	94.0 (93.3–94.7)	0.112
3-year	83.2 (82.2–84.3)	79.1 (71.2–85.1)	83.4 (82.3–84.4)	0.202
5-year	74.1 (72.8–75.4)	69.8 (61.0–77.0)	74.2 (72.9–75.6)	0.164
10-year	55.3 (53.3–57.1)	44.7 (32.7–56.0)	55.6 (53.6–57.5)	0.028
*Disease-free survival rate (%)*				
1-year	82.2 (81.1–83.2)	82.8 (75.4–88.1)	82.2 (81.0–83.2)	0.313
3-year	70.4 (69.1–71.7)	66.4 (57.4–73.9)	70.5 (69.2–71.8)	0.483
5-year	63.0 (61.5–64.5)	61.3 (51.9–69.3)	63.1 (61.5–64.5)	0.339
10-year	51.9 (49.9–53.8)	37.5 (25.3–49.7)	52.3 (50.3–54.2)	0.103

**Fig. 3:**
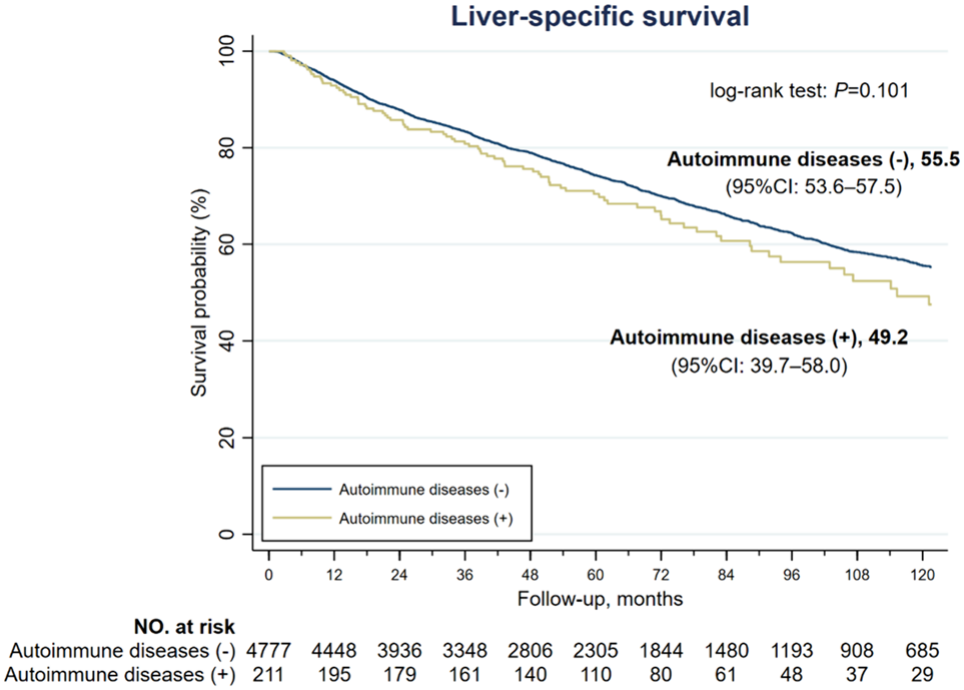
Kaplan-Meire liver-specific survival curves of HCC patients without and without autoimmune diseases. The median liver-specific survival was 49.2 months in the AD (+) group and 55.5 months in the AD (–) group (*P* = 0.101)

**Fig. 4:**
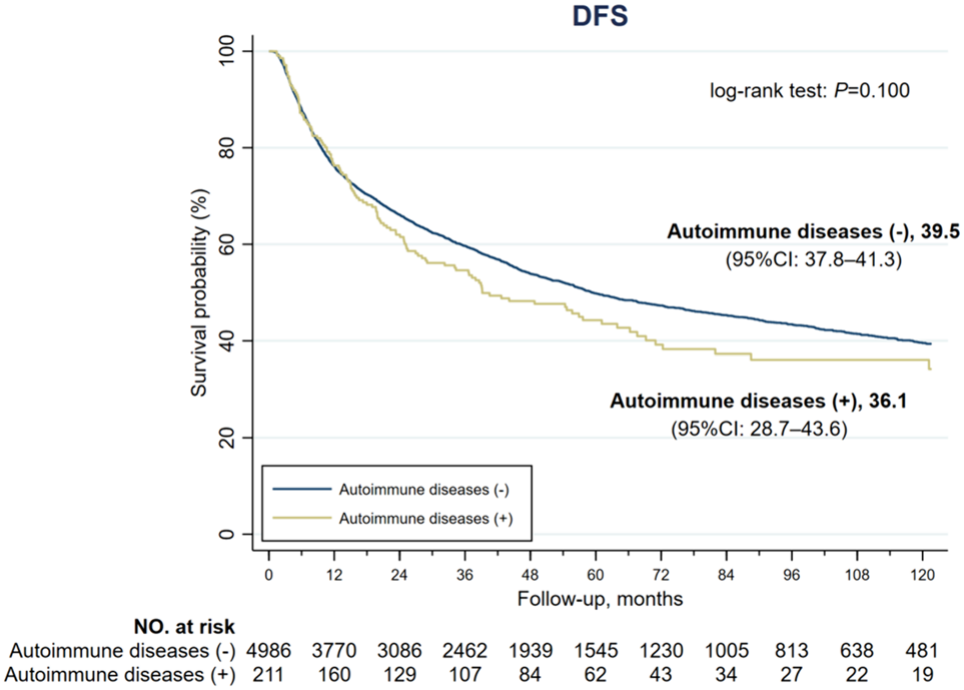
Kaplan-Meire disease-free survival (DFS) curves of HCC patients without and without autoimmune diseases. The median DFS was 36.1 months in the AD (+) group and 39.5 months in the AD (–) group (*P* = 0.100)

As for liver-specific survival, the estimated survival was comparable between AD (+) and AD (–) groups (49.2 vs. 55.5 months, *P* = 0.101), as shown in Fig. 3. The 1-year, 3-year and 5-year liver-specific survival rates were 93.3%, 83.2% and 74.1%, respectively, in the entire cohort and were comparable between the two groups. The 10-year liver-specific survival rate was 55.3% in the entire cohort and was significantly less in AD (+) than in AD (–) group (44.7% vs. 55.6%, *P* = 0.028) (Table 3). The estimated DFS was 36.1 months and 39.5 months in AD (+) and AD (–) groups, respectively, with a *P* value of 0.100 (Fig. 4). The 1-year, 3-year, 5-year and 10-year DFS rates were all comparable between the two groups (Table 3).

### Risk factors for mortality and tumor recurrence

Risk factors for all-cause mortality among HCC patients after liver resections were demonstrated in Table 4. After Cox regression multivariate analysis, the presence of AD did not lead to a higher risk of all-cause mortality (HR 0.93, 95% CI 0.59–1.47, *P* = 0.750). In fact, the significant prognostic factors for higher mortality were age greater than ≥ 65 years (HR 1.55), cirrhosis (HR 1.46), larger tumor size (HR 1.09 per 1 cm increase), advanced tumor stage (HR 1.61, 3.45, and 3.49 for stage II, III, and IV, respectively), α-fetoprotein (AFP) greater than 400 ng/ml (HR 1.25), and hemoglobin ≤ 10 g/dL (HR 1.54). Risk factors for liver-specific mortality were outlined in Table 5, which has shown similar results to all-cause mortality. In Table 6, the risks of tumor recurrence were higher among patients who were older than 65 years (HR 1.48), with liver cirrhosis status (HR 1.44), larger tumor size (HR 1.09 per 1 cm increase), and advanced tumor stage (HR 1.68 and 3.67 for stage II and III, respectively). The risks were also significantly elevated in patients with AFP level greater than 400 ng/ml (HR 1.36). The presence of AD did not increase the risk of tumor recurrence after liver resections (HR 1.00, 95% CI 0.60–1.67, *P* = 0.990). Subgroup analysis further validated that the presence of ADs in HCC patients rendered no significant adverse impact on liver-specific mortality nor recurrence, regardless of age, cirrhosis, serum AFP level, hemoglobin level and tumor size (Tables 7 and 8).

**Table 4:** Risk factors for all-cause mortality among operated hepatocellular carcinoma (HCC) patients

	Univariate		Multivariate	
	HR (95% CI)	*P*	HR (95% CI)	*P*
Autoimmune diseases	1.30 (1.03, 1.64)	0.025	0.93 (0.59, 1.47)	0.750
Male vs. female	1.16 (1.05, 1.29)	0.005	1.23 (0.99, 1.53)	0.060
Aged ≥ 65 vs. < 65 years	1.46 (1.34, 1.59)	< 0.001	1.55 (1.29, 1.86)	< 0.001
Diabetes	1.20 (1.09, 1.32)	< 0.001	1.23 (0.99, 1.53)	0.060
Hypertension	1.04 (0.95, 1.14)	0.390	0.87 (0.72, 1.05)	0.145
Chronic hepatitis	0.76 (0.70, 0.84)	< 0.001	1.00 (0.81, 1.23)	0.983
Cigarette smoking	1.14 (0.99, 1.31)	0.070	1.08 (0.86, 1.36)	0.488
Alcohol consumption	1.11 (0.95, 1.30)	0.176	1.05 (0.84, 1.32)	0.665
Betel nut chewing	1.22 (0.94, 1.57)	0.127	1.24 (0.88, 1.75)	0.213
Child-Pugh B vs. A	2.45 (1.73, 3.47)	< 0.001	1.34 (0.88, 2.04)	0.166
Cirrhosis vs. non cirrhosis	1.28 (1.12, 1.47)	< 0.001	1.46 (1.22, 1.75)	< 0.001
Tumor size per 1 cm increase	1.05 (1.04, 1.05)	< 0.001	1.09 (1.06, 1.11)	< 0.001
*TNM stage*				
II vs. I	1.64 (1.47, 1.82)	< 0.001	1.61 (1.30, 2.01)	< 0.001
III vs. I	3.55 (3.20, 3.94)	< 0.001	3.45 (2.71, 4.41)	< 0.001
IV vs. I	6.48 (4.91, 8.54)	< 0.001	3.49 (2.15, 5.66)	< 0.001
Anti-HCV/HBV therapy	0.58 (0.47, 0.71)	< 0.001	0.91 (0.67, 1.24)	0.543
Metformin	1.18 (0.99, 1.40)	0.063	1.18 (0.83, 1.69)	0.355
Aspirin	1.21 (0.99, 1.48)	0.062	1.21 (0.83, 1.75)	0.319
α-fetoprotein ≥ 400 vs. < 400	1.50 (1.34, 1.68)	< 0.001	1.25 (1.03, 1.51)	0.025
Albumin ≤ 3.5 vs. > 3.5	1.74 (1.56, 1.94)	< 0.001	0.83 (0.62, 1.11)	0.212
Hemoglobin ≤ 10 vs. > 10	1.82 (1.55, 2.14)	< 0.001	1.54 (1.14, 2.07)	0.005
Platelet ≤ 100 vs. > 100	1.32 (1.15, 1.51)	< 0.001	1.32 (0.96, 1.82)	0.084
INR > 1.4 vs. ≤ 1.4	1.68 (1.21, 2.34)	0.002	1.14 (0.65, 2.02)	0.650
AST > 102 vs. ≤ 102	1.54 (1.36, 1.74)	< 0.001	1.15 (0.84, 1.59)	0.382
ALT > 108 vs. ≤ 108	1.24 (1.09, 1.40)	0.001	0.86 (0.61, 1.21)	0.396
Total bilirubin > 1.5 vs. ≤ 1.5	1.49 (1.29, 1.74)	< 0.001	1.12 (0.83, 1.52)	0.463
PNI group (mod/severe vs. normal/mild)	1.71 (1.55, 1.89)	< 0.001	1.15 (0.88, 1.51)	0.294
ALBI group II/III vs. I	1.73 (1.58, 1.90)	< 0.001	1.19 (0.94, 1.50)	0.144
NLR every increase 1 unit	1.01 (1.00, 1.02)	0.003	0.98 (0.96, 1.00)	0.024
PLR every increase 1 unit	0.99 (0.98, 1.00)	0.005	1.00 (0.99, 1.02)	0.740

**Table 5:** Risk factors for liver-specific mortality among operated hepatocellular carcinoma (HCC) patients

	Univariate		Multivariate	
	HR (95% CI)	*P*	HR (95% CI)	*P*
Autoimmune diseases	1.35 (1.03, 1.76)	0.028	0.99 (0.60, 1.64)	0.977
Male vs. female	1.17 (1.04, 1.33)	0.010	1.16 (0.91, 1.48)	0.234
Aged ≥ 65 vs. < 65 years	1.38 (1.25, 1.53)	< 0.001	1.32 (1.08, 1.62)	0.008
Diabetes	1.18 (1.05, 1.32)	0.004	1.07 (0.83, 1.38)	0.579
Hypertension	1.03 (0.92, 1.14)	0.634	0.89 (0.72, 1.09)	0.261
Chronic hepatitis	0.81 (0.72, 0.90)	< 0.001	1.04 (0.83, 1.32)	0.711
Cigarette smoking	1.25 (1.07, 1.46)	0.005	1.06 (0.83, 1.36)	0.644
Alcohol consumption	1.27 (1.08, 1.51)	0.005	1.08 (0.84, 1.39)	0.531
Betel nut chewing	1.44 (1.11, 1.89)	0.007	1.31 (0.91, 1.89)	0.145
Child-Pugh B vs. A	2.57 (1.77, 3.73)	< 0.001	1.47 (0.96, 2.27)	0.078
Cirrhosis vs. non cirrhosis	1.33 (1.14, 1.54)	< 0.001	1.54 (1.26, 1.88)	< 0.001
Tumor size per 1 cm increase	1.06 (1.05, 1.06)	< 0.001	1.08 (1.06, 1.11)	< 0.001
*TNM stage*				
II vs. I	1.76 (1.56, 2.01)	< 0.001	1.65 (1.28, 2.12)	< 0.001
III vs. I	4.12 (3.65, 4.65)	< 0.001	3.95 (3.01, 5.17)	< 0.001
IV vs. I	7.94 (5.88, 10.74)	< 0.001	3.63 (2.12, 6.21)	< 0.001
Anti-HCV/HBV therapy	0.65 (0.52, 0.80)	< 0.001	0.98 (0.70, 1.37)	0.922
Metformin	1.23 (1.01, 1.50)	0.038	1.38 (0.92, 2.06)	0.121
Aspirin	1.18 (0.93, 1.51)	0.178	1.30 (0.86, 1.97)	0.206
α-fetoprotein ≥ 400 vs. < 400	1.51 (1.32, 1.72)	< 0.001	1.27 (1.03, 1.57)	0.026
Albumin ≤ 3.5 vs. > 3.5	1.79 (1.58, 2.04)	< 0.001	0.90 (0.65, 1.25)	0.535
Hemoglobin ≤ 10 vs. > 10	1.86 (1.54, 2.25)	< 0.001	1.61 (1.16, 2.23)	0.004
Platelet ≤ 100 vs. > 100	1.33 (1.14, 1.57)	< 0.001	1.36 (0.95, 1.94)	0.092
INR > 1.4 vs. ≤ 1.4	1.65 (1.12, 2.43)	0.012	0.95 (0.50, 1.80)	0.867
AST > 102 vs. ≤ 102	1.61 (1.40, 1.86)	< 0.001	1.05 (0.74, 1.48)	0.790
ALT > 108 vs. ≤ 108	1.30 (1.13, 1.50)	< 0.001	0.94 (0.65, 1.36)	0.752
Total bilirubin > 1.5 vs. ≤ 1.5	1.60 (1.35, 1.90)	< 0.001	1.22 (0.88, 1.69)	0.236
PNI group (mod/severe vs. normal/mild)	1.70 (1.51, 1.91)	< 0.001	0.99 (0.74, 1.33)	0.945
ALBI group II/III vs. I	1.82 (1.63, 2.03)	< 0.001	1.24 (0.96, 1.60)	0.094
NLR every increase 1 unit	1.01 (1.00, 1.02)	0.002	0.99 (0.97, 1.01)	0.331
PLR every increase 1 unit	1.00 (0.99, 1.00)	0.251	1.01 (0.99, 1.03)	0.180

**Table 6:** Risk factors for tumor recurrence among operated hepatocellular carcinoma (HCC) patients

	Univariate		Multivariate	
	HR (95% CI)	*P*	HR (95% CI)	*P*
Autoimmune diseases	1.23 (0.96, 1.59)	0.104	1.00 (0.60, 1.67)	0.990
Male vs. female	1.15 (1.02, 1.28)	0.017	1.14 (0.90, 1.42)	0.272
Aged ≥ 65 vs. < 65 years	1.47 (1.34, 1.61)	< 0.001	1.48 (1.22, 1.79)	< 0.001
Diabetes	1.10 (0.99, 1.23)	0.066	1.22 (0.98, 1.53)	0.078
Hypertension	1.00 (0.91, 1.10)	1.000	0.86 (0.70, 1.04)	0.120
Chronic hepatitis	0.84 (0.76, 0.93)	0.001	1.10 (0.88, 1.37)	0.395
Cigarette smoking	1.00 (0.86, 1.17)	0.955	1.16 (0.91, 1.48)	0.239
Alcohol consumption	0.94 (0.79, 1.10)	0.430	0.99 (0.77, 1.26)	0.907
Betel nut chewing	1.12 (0.86, 1.47)	0.398	1.44 (1.00, 2.05)	0.047
Child-Pugh B vs. A	2.43 (1.64, 3.59)	< 0.001	1.37 (0.85, 2.20)	0.195
Cirrhosis vs. non cirrhosis	1.36 (1.18, 1.57)	< 0.001	1.44 (1.19, 1.74)	< 0.001
Tumor size per 1 cm increase	1.05 (1.04, 1.05)	< 0.001	1.09 (1.06, 1.11)	< 0.001
*TNM stage*				
II vs. I	1.69 (1.51, 1.89)	< 0.001	1.68 (1.34, 2.10)	< 0.001
III vs. I	3.51 (3.14, 3.94)	< 0.001	3.67 (2.84, 4.73)	< 0.001
IV vs. I	6.60 (4.46, 9.76)	< 0.001	2.25 (0.96, 5.34)	0.065
Anti-HCV/HBV therapy	0.56 (0.46, 0.69)	< 0.001	0.90 (0.65, 1.25)	0.543
Metformin	1.03 (0.85, 1.24)	0.781	1.01 (0.69, 1.49)	0.963
Aspirin	1.11 (0.89, 1.39)	0.347	1.39 (0.94, 2.06)	0.097
α-Fetoprotein ≥ 400 vs. < 400	1.54 (1.37, 1.74)	< 0.001	1.36 (1.11, 1.67)	0.003
Albumin ≤ 3.5 vs. > 3.5	1.72 (1.52, 1.93)	< 0.001	0.95 (0.70, 1.30)	0.756
Hemoglobin ≤ 10 vs. > 10	1.59 (1.33, 1.91)	< 0.001	1.30 (0.93, 1.82)	0.128
Platelet ≤ 100 vs. > 100	1.48 (1.28, 1.71)	< 0.001	1.31 (0.95, 1.82)	0.102
INR > 1.4 vs. ≤ 1.4	1.47 (0.99, 2.16)	0.053	0.80 (0.40, 1.59)	0.519
AST > 102 vs. ≤ 102	1.60 (1.40, 1.83)	< 0.001	0.93 (0.66, 1.31)	0.687
ALT > 108 vs. ≤ 108	1.37 (1.20, 1.57)	< 0.001	1.09 (0.77, 1.56)	0.620
Total bilirubin > 1.5 vs. ≤ 1.5	1.49 (1.27, 1.76)	< 0.001	1.09 (0.78, 1.53)	0.595
PNI group (mod/severe vs. normal/mild)	1.72 (1.54, 1.91)	< 0.001	1.04 (0.78, 1.38)	0.785
ALBI group II/III vs. I	1.77 (1.60, 1.95)	< 0.001	1.26 (0.99, 1.62)	0.064
NLR every increase 1 unit	1.01 (1.00, 1.02)	0.003	0.98 (0.96, 1.00)	0.024
PLR every increase 1 unit	0.99 (0.98, 1.00)	0.005	1.00 (0.99, 1.02)	0.740

**Table 7:** Subgroup analysis on risks of liver-specific mortality among operated hepatocellular carcinoma (HCC) patients

Subgroup	Autoimmune		Adjusted HR		Wald test
	(+)	(–)	(95% CI)	*P*	*P*
Age, years					0.861
< 65	33/88	955/3,295	0.92 (0.43–1.98)	0.827	
≥ 65	23/48	562/1,557	1.00 (0.50–1.98)	0.990	
Cirrhosis					0.405
No	9/36	303/1,506	0.73 (0.29–1.86)	0.510	
Yes	15/44	369/1,401	1.19 (0.65–2.20)	0.568	
Serum AFP					0.923
≤ 400ng/mL	32/92	853/2,969	0.93 (0.53–1.65)	0.804	
> 400ng/mL	7/17	295/745	0.85 (0.26–2.72)	0.780	
Hemoglobin					0.999
< 10 g/dL	39/104	1,211/3,996	1.03 (0.58–1.81)	0.924	
≥ 10 g/dL	11/19	105/230	1.22 (0.36–4.10)	0.750	
Tumor size					0.713
< 6.5 cm	41/108	975/3,772	1.11 (0.61–2.04)	0.731	
≥ 6.5 cm	15/28	542/1,080	0.77 (0.30–2.02)	0.600	
Tumor size					0.752
< 5 cm	37/99	795/3,250	1.07 (0.55–2.08)	0.842	
≥ 5 cm	19/37	722/1,602	0.78 (0.35–1.74)	0.545	

**Table 8:** Subgroup analysis on risks of tumor recurrence among operated hepatocellular carcinoma (HCC) patients

Subgroup	Autoimmune		Adjusted HR		Wald test
	(+)	(–)	(95% CI)	*P*	*P*
Age, years					0.992
< 65	38/93	1049/3356	0.98 (0.45–2.14)	0.964	
≥ 65	24/49	727/1702	0.93 (0.47–1.85)	0.834	
Cirrhosis					0.201
No	8/35	335/1511	0.65 (0.23–1.82)	0.409	
Yes	17/46	401/1416	1.26 (0.69–2.32)	0.449	
Serum AFP					0.998
≤ 400ng/mL	37/97	1018/3104	1.00 (0.57–1.77)	0.997	
> 400ng/mL	8/18	334/770	0.83 (0.23–2.94)	0.772	
Hemoglobin					0.236
< 10 g/dL	46/111	1428/4167	1.27 (0.75–2.14)	0.376	
≥ 10 g/dL	8/16	122/245	0.27 (0.03–2.35)	0.237	
Tumor size					0.395
< 6.5 cm	48/115	1191/3954	1.11 (0.61–2.01)	0.732	
≥ 6.5 cm	14/27	585/1104	0.58 (0.21–1.64)	0.306	
Tumor size					0.713
< 5 cm	45/107	977/3406	1.10 (0.60-2.00)	0.765	
≥ 5 cm	17/35	799/1652	0.54 (0.19–1.50)	0.236	

## Discussion

Autoimmune diseases and cancer are the two debilitating situations that involve deranged inflammatory process. AD affect 5–10% ^35^of the population worldwide with female preponderance and is characterized by the production of autoantibodies that involve interactions between T- and B-cells and their subsets. Encompassing proinflammatory and anti-inflammatory mechanisms, almost all body parts can be affected by AD. The exact mechanism is still unclear, but intrinsic and environmental factors such as genetic, hormonal, stress, infections and drugs have been suggested (Anaya et al. 2016). Therapy for AD traditionally involves steroids and disease modifying antirheumatic drugs (DMARDs) that nonspecifically target inflammation whereas newer treatments involve targeted immunotherapy against B-, T-cells, co-stimulatory mediators and intracellular kinases (Jung et al. 2022). Interestingly, AD have been reported to exert positive and negative associations to cancer. For example, patients with SLE are at higher risks of hematologic malignancies such as non-Hodgkin’s lymphoma and leukemia and solid tumors including thyroid, liver, gallbladder, kidney and cervix have been reported. Patients with RA are also at an increased risk of aforementioned cancers in addition to lung cancer (Giat et al. 2017; Zhou 2022). That said, no difference in survival has been demonstrated in patients with AD and lung cancer (Jacob et al. 2020). On the other hand, Wadstrom et al. have demonstrated a reduced risk of incident breast cancer in women with RA (Wadström et al. 2020). However, despite numerous efforts and reports, few studies to date had tried to explore the potential influence of AD on the survival of HCC patients undergoing hepatectomies. Our study, as a result, is by far one of the largest series in the English literature to investigate the prognosis of AD patients after liver resections for HCC.

Management of cancer in patients with AD is complex and requires a multidisciplinary approach. Primary liver cancer is the 6th most common cancer globally and the 3rd leading cause of cancer related mortality, among which, HCC is the most common type, constituting 75–86% of cases (Sung et al. 2021; Ferlay et al. 2021). The major risk factor for HCC is liver cirrhosis secondary to alcohol abuse and viral hepatitis and also non-alcoholic fatty liver disease (NAFLD) in the absence of cirrhosis. Currently, the treatment options for HCC patients include surgical, locoregional and systemic therapies. Surgical resection is the curative treatment of choice for localized HCC, followed by routine postoperative surveillance for recurrence as the risk of recurrence remains high in the first year after resection (Tabrizian et al. 2015). Local ablative therapies such as thermal ablation, radiation segmentectomy and external beam radiation therapy (EBRT) have likewise been suggested for patients with HCC who are not eligible for surgery. Trans-arterial chemoembolization (TACE) and trans-arterial radioembolization (TARE) are two other options of HCC treatments, given a lower risk of hepatic dysfunction. Recently, systemic therapies have been approved in the treatment of HCC and they fall into two categories. Anti-angiographic targeted therapies include multi-target tyrosine kinase inhibitors (TKI) and monoclonal antiangiogenic antibodies while immune checkpoint inhibitors (ICI) include inhibitors of programmed death 1 (PD1) or its ligand (PD-L1) and cytotoxic T lymphocyte-associated protein 4 (CTLA4) inhibitors. Of the two categories, ICI are often well tolerated with superior survival outcome and are endorsed by major guidelines to become the first line systemic therapy for advanced HCC (Singal et al. 2023; Kudo et al. 2022). Moreover, due to superior recurrence-free survival, the American Association for the Study of Liver Diseases (AASLD) has recommended use of adjuvant ICI-based systemic therapy in patients at high risk of recurrence after hepatectomy or local ablation. Even more, ICI-based conversion therapy has been proposed as a promising treatment concept to either increase the resectability or reduce the post-operative tumor recurrence (Kudo et al. 2023; Hsu et al. 2023). Nevertheless, since most clinical trials have not demonstrated the safe and effective use of ICI in patients with AD and the REISAMIC registry has found an exacerbation of irAE with the use of ICI, ICI are not recommended in patients with AD or potential transplant candidates (Singal et al. 2023; Rimassa et al. 2021). The treatment alternatives for patients with AD are thus limited.

In our study, we have presented that the overall survival and liver-specific survival after hepatectomies are comparable in patients with and without autoimmune diseases. The risk factors for overall mortality and liver-specific mortality are old age, cirrhosis, larger tumor size, higher TNM staging, higher serum AFP level and anemia, irrespective of the presence of AD. Additionally, the current study also demonstrated that the DFS is comparable between patients with and without AD. That say, patients with AD are not at high risk for recurrence after hepatectomy and could be monitored routinely as those without AD. Based on these findings, we have some suggestions for patients with AD. First, for early-stage HCC, such as those with smaller tumor size, without major vascular invasion, and distant from vital vascular /biliary structures, upfront liver resection can be considered if the AD is under maintenance therapy or in stable state. From the current study, these patients can enjoy a long-term survival similar to those without AD. For more advanced HCC, such as those with large tumor size or Vp1/Vp2 vascular invasion, either non-ICI-based neoadjuvant treatments or upfront surgery are both justified. Rheumatological consultation prior to initiation of cancer treatment are also mandatory. In short, with careful patient selection by experienced liver surgeons, surgical resections may in fact provide similar results to patients with AD and should be considered in patients eligible for liver resections. Further studies are warranted to verify our findings.

Consistent with the literature characterizing female gender as a major genetic factor for AD, AD occurred with a higher frequency in women than men in our HCC population. The pattern may relate to the actions of estrogens and progestins, both of which account for the heightened immunological response to infections and vaccinations (Davidson et al. 2001; Pisetsky and Pisetsky 2023). AD were also associated with higher risks for hypertension, cardiovascular diseases, diabetes and hepatitis in consistency to the literature (Wolf et al. 2019; Wang et al. 2015). In other words, the patient population presented in the current study was not biasly selected and should reflect the real-world scenario.

Despite remarkable findings, the current study still has several limitations. First, although the median OS and liver-specific survival were comparable between the two groups, the 10-year OS rate and liver-specific survival rate were reduced in patients with AD. We believe this can be attributed to the influence of AD, i.e., the patients may eventually die from AD, or the AD per se or its related treatments may worsen the underlying hepatitis/cirrhosis. Further studies are still necessary to prove our hypothesis. Secondly, encompassing few patients with AD and a wide array of diseases, subgroup analysis of AD was not further performed and thus it was difficult to interpret the interactions between different AD and the survival outcome. Third, although the patient population was consistent with the literature, liver surgeons in CGMHs would only operate on those AD patients in stable disease or undergoing maintenance therapy. That is, those with active disease flare up would be excluded from liver resections. The feasibility and safety of hepatectomies in patients with disease flare up thus remain uncertain and deserve further investigations. Next, the current study was generated from the hospital-based database and cancer registry, more descriptive variables such as performance status, postoperative complications, and pathologic details, for example margin status, hepatitis activity index, and histological grade, were inaccessible. The analysis of these variables was thus lacking. Moreover, although the potential recall bias could be avoided by prospectively registering the daily clinical data into the CGRD, referral bias was in the meanwhile inevitable since the CGMHs are the largest tertiary care center in Taiwan (Sedgwick and Sedgwick 2012; Sedgwick and Sedgwick 2014). Lastly, since the current research was based primarily on data from a single country, the patient population thus would be rather uniform. The lack of different ethnic groups would be another drawback of the current study. It would be more solid if HCC datasets from countries with a more diverse population can be incorporated into the current research. Further studies are thus warranted to validate our findings.

## Conclusion

In conclusion, our CGRD-based multi-institutional study demonstrated that autoimmune disease does not impair the OS and DFS of HCC patients undergoing liver resections. AD itself is not a risk factor for tumor recurrence after surgery. Patients eligible for liver resections, as a result, should be considered for surgery irrespective of the presence of AD. However, the lack of data concerning liver resections in patients with AD flare up also warrants additional studies. Further research is thus mandatory to validate our findings.

## Data Availability

All data generated during this study are available from the corresponding author upon reasonable request.

## Abbreviations

AASLD: American Association for the Study of Liver Diseases
AD: Autoimmune diseases
AFP: α-fetoprotein
AIH: Autoimmune hepatitis
ALT: Alanine transaminases
AST: Aspartate transaminases
CGMH: Chang Gung Memorial Hospital
CGRD: Chang Gung Research Database
CI: Confidence interval
CTLA4: Cytotoxic T lymphocyte-associated protein 4
DFS: Disease-free survival
DM: Dermatomyositis
DMARD: Disease modifying antirheumatic drugs
EBRT: External beam radiation therapy
HBV: Hepatitis B virus
HCC: Hepatocellular carcinoma
HCV: Hepatitis C virus
HR: Hazard ratio
ICD-9-CM: International Classification of Diseases, 9th revision, Clinical Modification
ICD-10-CM: International Classification of Diseases, 10th revision, Clinical Modification
HR: Hazard ratio
ICD-O-3: International Classification of Diseases for Oncology, 3rd edition
ICIs: Immune checkpoint inhibitor
irAE: Immune-related adverse events
INR: International normalized ratio
NLR: Neutrophil to lymphocyte ratio
NAFLD: Non-alcoholic fatty liver disease
OS: Overall survival
PBC: Primary biliary cholangitis
PD-1: Programmed death 1
PD-L1: Programmed death 1 ligand
PLR: Platelet to lymphocyte ratio
PM: Polymyositis
PNI: Prognostic nutritional index
PSC: Primary sclerosing cholangitis
RA: Rheumatoid arthritis
SLE: Systematic lupus erythematosus
TACE: Trans-arterial chemoembolization
TARE: Trans-arterial radioembolization
